# The Spectrum of Clinical Characteristics and Complications of Tetanus: A Retrospective Cross-Sectional Study From a Developing Nation

**DOI:** 10.7759/cureus.15484

**Published:** 2021-06-06

**Authors:** Talal Almas, Muhammad Ali Niaz, Syed Muhammad Jawad Zaidi, Mohammad Haroon, Tarek Khedro, Reema Alsufyani, Abdulla Hussain Al-Awaid, Estelle Tran, Abdul Wali Khan, Hasan Alaeddin, Ali Rifai, Kuvira T Manamperi, Abat Khan, Abdul Haadi

**Affiliations:** 1 Internal Medicine, Royal College of Surgeons in Ireland, Dublin, IRL; 2 Surgery, Royal College of Surgeons in Ireland, Dublin, IRL; 3 Internal Medicine, Rawalpindi Medical University, Rawalpindi, PAK; 4 Internal Medicine, Khyber Teaching Hospital, Peshawar, PAK; 5 Internal Medicine, College of Physicians and Surgeons Pakistan, Peshawar, PAK; 6 Internal Medicine, Hayatabad Medical Complex, Peshawar, PAK; 7 Cardiology, Khyber Teaching Hospital, Peshawar, PAK; 8 Internal Medicine, Royal College of Surgeons In Ireland, Dublin, IRL

**Keywords:** infectious diseases, tetanus, vaccine, respiratory failure, clostridium tetani

## Abstract

Introduction

While tetanus has largely been eradicated with the advent of the tetanus vaccine, its prevalence in Pakistan remains alarmingly high due to insufficient uptake of the vaccination program. The clinical presentations that the disease elicits range from mere opisthotonos to more sinister complications, including respiratory failure and death, often posing an insurmountable challenge for hospitals.

Methods

A retrospective cross-sectional study was conducted and analyzed the medical charts of 43 patients with a confirmed diagnosis of tetanus infection. The charts were perused for the patients’ demographics, clinical characteristics, and disease outcomes. The prevalence of various clinical symptoms and complications were reported in terms of frequencies and percentages.

Results

The mean age of the patients hovered at 29.53 ± 16.53 years, with a range of 12 to 65 years. Of those affected, 83.7% were males while 16.3% were females. Notably, none of the infected patients had a prior history of vaccination against tetanus. Trismus was noted to be the most prevalent clinical manifestation and was found in 90.70% of the patients while paraesthesia at the site of infection, found in 6.98%, was the least prevalent. The overall mortality was noted to hover at 46.5%.

Conclusion

While tetanus has largely been eradicated, its prevalence in Pakistan remains alarmingly high. The complications noted in the study have implications for the country’s public health system and aims to better inform the current state of the national vaccination program.

## Introduction

Tetanus is a potentially life-threatening infectious disease caused by the spore-forming, gram-positive bacilli Clostridium Tetani. This organism’s spores reside in environmental soil and can penetrate through a defect in the mechanical skin barrier sequentially to a wound injury [1]. Geographically, it is most prevalent in developing countries due to a lack of immunization or failure to receive a booster dose in the 10 years following the prior series of vaccinations [2]. This anaerobic bacterium produces tetanospasmin, an endotoxin that prevents the release of neurotransmitters that inhibit muscular contractions, thereby leading to spontaneous muscle spasms and overall body rigidity. Clinically, the illness manifests with features of hypertonia characterized by lockjaw (trismus), a sneering grin facial expression (rhisus sardonicus), involuntary muscle contractions associated with pain, and involuntary urination and defecation [3,4]. In more severe cases, dysphagia can be present as a consequence of pharyngeal muscle spasms. Back arching spasms, known as opisthotonos, leg extensions, arm flexions with episodes of apnea as a result of abdominal, diaphragmatic and intercostal muscles rigidity can also be noted. Other possible symptoms include fever, hypertension, tachycardia and sweating [4]. Tetanus has four distinct classifications: neonatal, which usually occurs in infants born to unimmunized mothers; cephalic, which often follows a head injury or an acute otitis media affecting the cranial nerves; localized, which is persistent muscle spasms at the site of injury; and generalized, which involves all parts of the body. The predominant complications of this possibly fatal ailment are attributable to contractions of the respiratory muscles, consequently leading to respiratory arrest. Others include upper airway obstruction, aspiration pneumonia, long bone fractures, cardiac arrhythmias, coma, and seizures, which can all culminate in significant morbidity and mortality [4,5]. Management of an ongoing tetanus infection depends on the severity of the disease, but the overarching aims of treatment are neutralization of the tetanus toxin with human tetanus immunoglobulin, wound debridement for spore eradication, control of disease manifestations, and management of further complications [3]. Additional treatment measures involve antibiotic therapy, mechanical ventilation, and high-calorie nutritional support, which can curtail the disease duration [5]. The overarching aim of the present study is to elucidate the diversity in clinical presentations of the disease and its complications and outcomes. 

## Materials and methods

This retrospective cross-sectional study involved a total of 43 patients clinically diagnosed with tetanus infection due to wound injury who were admitted to our hospital. The patients were studied for various parameters including their demographics, clinical characteristics, and disease outcomes. The prevalence of various clinical symptoms and complications was reported in terms of frequencies and percentages. Tetanus patients with multiple comorbidities, such as those suffering from end-stage systemic illnesses and cancer, were excluded from the study. The exclusion criteria ensured the elimination of confounding factors in reporting the prevalence of various complications of tetanus. The association of various complications with survival status was cross-tabulated and analyzed using the Chi-square test. The normality of the data was assessed using the Shapiro Wilk test. Additionally, independent samples t-test and Mann Whitney U-test were employed to compare normally and non-normally distributed scale variables, respectively, with survival status. A p-value of less than 0.05 was considered statistically significant. Data were then analyzed using the Statistical Package for Social Sciences (SPSS), version 23.0 software (IBM Corporation, Armonk, NY).

## Results

In the present study involving 43 patients, the mean age of the study participants was 29.53 ± 16.53 years, with a range of 12 to 65 years. None of the patients were completely vaccinated against tetanus. The demographic details of the study participants are delineated in Table 1.

**Table 1:** The background characteristics of the study participants included.

Parameters		Frequency	Percentages
Gender	Male	36	83.7%
	Female	7	16.3%
Marital status	Married	26	60.5%
	Unmarried	17	39.5%
Immunization history	Partial immunization	3	6.9%
	No immunization	40	93.1%
Profession	Student	12	27.9%
	Carpenter	2	4.7%
	Farmer	12	27.9%
	Labourer	10	23.3%
	Shopkeeper	1	2.3%
	Unemployed	6	14%
Comorbidities	Diabetes mellitus	8	18.6%
	Hypertension	5	11.6%
	Stable angina	1	2.3%
	Chronic kidney disease	2	4.7%
	No comorbidities	27	62.8%

**Figure 1:**
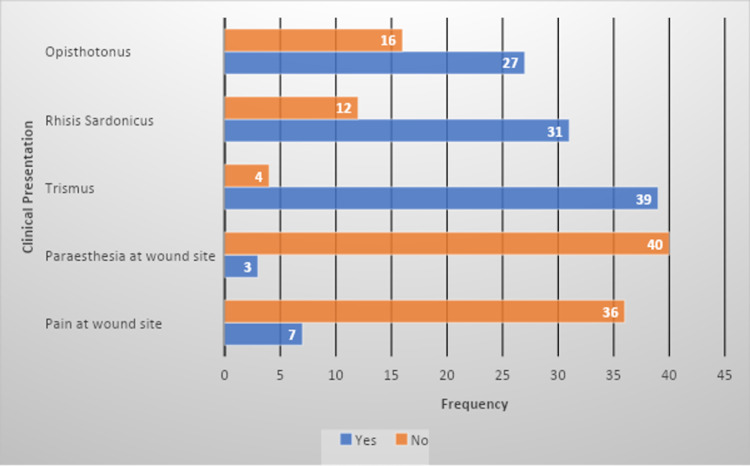
A depiction of the clinical presentation of the study participants.

Based on the clinical examination, history, and wound assessment, an initial diagnosis of tetanus was made. The patients presented with a plethora of different signs and symptoms as observed on history and clinical examination. Figure 1 further delineates the various clinical presentations of the patients that were included in the present study. 

Furthermore, details of the clinical symptoms of the patients are depicted in Table 2.

**Table 2:** A tabulation of the patients’ clinical symptoms and the associated prevalence.

Clinical symptoms		Frequency	Percentages
Dyspnea	Yes	18	41.9%
	No	25	58.1%
Tachycardia	Yes	29	67.4%
	No	14	32.6%
Cranial nerves involvement	Yes	2	4.7%
	No	41	95.35
Dysphagia	Yes	29	67.4%
	No	14	32.6%
Labile blood pressure	Yes	15	34.9%
	No	28	65.1%
Urinary retention	Yes	13	30.2%
	No	30	69.8%
Constipation	Yes	25	58.1%
	No	18	41.9%
Type of tetanus	Localized	1	2.3%
	Generalized	42	97.7%

Pertinently, all patients were treated with intravenous antitetanic immunoglobulins and antibiotics in order to combat the ramifications of the infection. Additionally, wound cleaning and debridement were also performed as a part of management. Out of the 43 patients, 20 (46.5%) expired from complications of the illness, while the other 23 (53.5%) patients eventually recovered. Respiratory arrest and aspiration pneumonia were associated with mortality (p < 0.05). Table 3 cross-tabulates the survival status as it corresponds to the various disease complications.

**Table 3:** A cross-tabulation of the complications with the survival status as observed within the study participants. *p-value obtained using the Chi-square test.

Complications		Outcome		p-value*
		Cured (n = 23)	Expired (n = 20)	
Respiratory arrest	Yes	5	15	0.001
	No	18	5	
Aspiration pneumonia	Yes	6	16	0.001
	No	17	4	
Need for ventilation	Yes	1	2	0.446
	No	22	18	

Furthermore, patients that expired from the disease had a shorter time to the onset of symptoms after wound injury (in days) than those who were cured. The comparison of other study variables is elucidated in Table 4.

**Table 4:** A comparison of the survival status with the time of onset and the incubation period. *Mann Whitney U test. **Independent samples t-test. Normality of the data was assessed using the Shapiro Wilk test.

Parameter	Survival status	Median (range)/mean ± standard deviation	p-value
Time of onset (in days)	Expired	2.00 (1.00-8.00)	0.003*
	Cured	4.00 (0.00-16.00)	
Incubation period (in days)	Expired	11.20 ± 6.46	0.482**
	Cured	12.83 ± 8.52	

## Discussion

Tetanus is a disease caused by infection with Clostridium tetani, a gram-positive anaerobic rod-shaped bacterium [6]. It is found in environmental soil throughout the world and infects through wounds. However, in around 20% of cases, no identifiable wound is present [7]. Once inside its host, the bacteria produce the tetanus toxin that inhibits the soluble N-ethylmaleimide-sensitive factor (NSF) attachment protein, the synaptosomal-associated protein (SNAP) receptor, of the soluble NSF attachment protein receptor (SNARE) complex, ultimately preventing the release of inhibitory neurotransmitters in neurons [8]. This leads to the classic clinical presentation of tetanus characterized by mild muscle spasms that, if left untreated, progress to severe prolonged spasms and resultant respiratory failure. The characteristic spasms include risus sardonicus, trismus (“lockjaw”), and opisthotonos. The current retrospective study examined the clinical characteristics, progression, and outcomes of 43 patients with a confirmed tetanus infection. The diagnosis was made after a meticulous history, clinical examination, and laboratory workup of patients who had sustained a wound injury. Out of the 43 patients, 42 (97.7%) developed generalized tetanus, and nonspecific symptoms reported included dysphagia (67.4%), tachycardia (67.4%), constipation (58.1%), dyspnea (41.9%), labile blood pressure (34.9%), urinary retention (30.2%), and cranial nerve involvement (4.7%). Other more tetanus-specific clinical signs that were observed included trismus (90.7%), risus sardonicus (72.1%), opisthotonos (62.8%), pain at the wound site (16.3%), and paraesthesia at the wound site, which was observed in 6.98% of the patients. 

In one case of generalized tetanus, which involves descending muscle spasms, with a physical examination positive for masseter muscle stiffness and dysphagia, the patient was administered a dose of intramuscular human tetanus immunoglobulin, tetanus toxoid vaccine, and ceftriaxone (2,000 mg loading dose followed by 1,000 mg Q12H) and metronidazole (500 mg Q6H) [9]. In two studies that compared the efficacy of penicillin to metronidazole in tetanus treatment, metronidazole was shown to significantly outperform the penicillin in preventing progression to death [10,11]. The first study reported a 24% (18/76 patients) mortality rate for procaine benzylpenicillin and 7% (7/97 patients) for metronidazole [10]. The second study found a 46% (26/56) mortality rate for IM benzathine benzylpenicillin, 44% (22/50) for IV benzylpenicillin, and 35% (19/55) for metronidazole [11]. While theoretical advantages of human antitoxin preparations when compared to equine preparations include longer half-lives and a reduced potential for hypersensitivity reactions, they are typically more expensive and also unavailable in some countries, which also often have a soaring incidence of tetanus [12]. This might be a potential explanation for the alarming mortality rates observed in our study. Similarly, the treatment for all 43 patients in this study included human antitetanic immunoglobulin, 500 mg metronidazole and 500 mg penicillin. As tetanus management also involves the prevention of toxin uptake, wound cleaning and debridement were also performed. Out of the 43 patients studied, 20 expired (46.5%). Respiratory arrest was present in 15 out of 20 patients who expired compared to merely five out of 23 in those who were cured. Respiratory compromise and failure therefore correlate very closely with mortality rates. Aspiration pneumonia was present in 80% of the patients who expired and in 26.1% of the patients who subsequently recovered. Not surprisingly, patients who developed respiratory complications were more likely to die. Furthermore, a shorter time of onset was associated with a higher mortality likelihood (two days in those expired versus 4 days in those cured). This is likely due to the shorter time of onset for severe symptoms that consequently lead to death. Imperatively, incubation period was not found to be a predictor of death.

Today, tetanus sees a relatively low incidence in the developed world largely due to the high rates of immunization against the tetanus toxin, tetanospasmin. However, this rapidly fatal disease remains a public health threat in developing countries throughout the world. Studies in 2015 estimated that 79% of deaths due to tetanus occurred in south Asia and sub-Saharan Africa [6]. Nevertheless, measuring its true impact on global health is difficult due to the limited surveillance systems in place in the countries it is most prevalent [6]. What is clear, however, is that despite the underreporting from the local health systems, the incidence of tetanus spikes when natural disasters strike [7]. In the past few decades, globalization has made for more stronger responses to disasters such as earthquakes. For example, doctors often travel to areas affected by disaster and provide aid to strained healthcare system. Unfortunately, the causes of higher incidences, outbreaks, and mortalities are multifactorial, and include low immunization rates, dwindling clinical awareness directed towards more apparent traumas, injuries sustained during disasters, limited medical supplies, and delay in treating an active tetanus infection [7].

Forty patients (93%) in the present study were not vaccinated against tetanospasmin. The remaining three were only partially vaccinated. These immunization rates are not dissimilar to those that would be seen in similar, developing countries across the world [12,13]. In fact, fewer than two-thirds of all countries achieved the Global Vaccine Action Plan (GVAP) 2020 target of 90% national coverage with the third dose of diphtheria and tetanus toxoid and pertussis vaccine (DTP3) [13]. Even in instances where patients are immunized, the dropout rates between the first and third dose in low-income countries (7%) are greater than middle- (4%) and high-income countries (3%) [12,13]. Further vaccination efforts are mandated to curb the incidence and burden of tetanus on societies. Lastly, public education is of the utmost importance. Most of the patients included did not present to the clinic after sustaining a wound injury. Instead, they presented after the onset of one of the more serious symptoms such as trismus. Education campaigns could curtail the time to presentation to the clinic after sustaining a wound that would allow for timely prophylaxis against tetanus.

## Conclusions

Tetanus is an infectious ailment that can culminate in a myriad of serious complications, including respiratory arrest and death. With the advent of the tetanus vaccine, the mortality rates in the western world have dramatically plummeted; however, in resource-deprived nations such as Pakistan, tetanus continues to be the cause of alarmingly high mortality rates. Public health campaigns aimed at promoting the uptake of an exhaustive vaccination regimen will be pivotal in curbing the infection rates in the future.
